# Effectiveness of Music Education for the Improvement of Reading Skills and Academic Achievement in Young Poor Readers: A Pragmatic Cluster-Randomized, Controlled Clinical Trial

**DOI:** 10.1371/journal.pone.0059984

**Published:** 2013-03-27

**Authors:** Hugo Cogo-Moreira, Clara Regina Brandão de Ávila, George B. Ploubidis, Jair de Jesus Mari

**Affiliations:** 1 Department of Psychiatry, Federal University of São Paulo, São Paulo, Brazil; 2 Department of Hearing and Speech Pathology, Federal University of São Paulo, São Paulo, Brazil; 3 Department of Population Studies, University of London, London School of Hygiene and Tropical Medicine, Faculty of Epidemiology and Population Health, London, United Kingdom; Federal University of Rio de Janeiro, Brazil

## Abstract

**Introduction:**

Difficulties in word-level reading skills are prevalent in Brazilian schools and may deter children from gaining the knowledge obtained through reading and academic achievement. Music education has emerged as a potential method to improve reading skills because due to a common neurobiological substratum.

**Objective:**

To evaluate the effectiveness of music education for the improvement of reading skills and academic achievement among children (eight to 10 years of age) with reading difficulties.

**Method:**

235 children with reading difficulties in 10 schools participated in a five-month, randomized clinical trial in cluster (RCT) in an impoverished zone within the city of São Paulo to test the effects of music education intervention while assessing reading skills and academic achievement during the school year. Five schools were chosen randomly to incorporate music classes (n = 114), and five served as controls (n = 121). Two different methods of analysis were used to evaluate the effectiveness of the intervention: The standard method was intention-to-treat (ITT), and the other was the Complier Average Causal Effect (CACE) estimation method, which took compliance status into account.

**Results:**

The ITT analyses were not very promising; only one marginal effect existed for the rate of correct real words read per minute. Indeed, considering ITT, improvements were observed in the secondary outcomes (slope of Portuguese = 0.21 [p<0.001] and slope of math = 0.25 [p<0.001]). As for CACE estimation (i.e., complier children versus non-complier children), more promising effects were observed in terms of the rate of correct words read per minute [β = 13.98, p<0.001] and phonological awareness [β = 19.72, p<0.001] as well as secondary outcomes (academic achievement in Portuguese [β = 0.77, p<0.0001] and math [β = 0.49, p<0.001] throughout the school year).

**Conclusion:**

The results may be seen as promising, but they are not, in themselves, enough to make music lessons as public policy.

## Introduction

Due to the demands of an increasingly technological society, reading failure has a major impact on cognitive development [1], [2]. Obtaining adequate reading comprehension of written material is the ultimate goal of reading, and achievement of word-level skills is used as an initial indicator of success in learning to read [3]. In 2009, Brazil was ranked 53^rd^ among 65 participating countries in reading and science achievement and 57^th^ in math via the Programme for International Student Assessment (PISA) by the Organization for Economic Co-Operation and Development. Though PISA analyzed 15-year-old children (an older population when compared with our sample of 8- to 10-year -olds), these indicators warrant attention from authorities not only in Brazil but also in other countries with low achievement (e.g., Peru, Panama, Montenegro, Bulgaria, and the Russian Federation).The most common approach to reading intervention has a theoretical motivation: Good phonological and metaphonological skills are important for success in learning to read. Children who have reading difficulties have deficits in these skills and training in phonological skills in the context of reading has repeatedly been shown to lead to improvement in reading, at least in English [4].

Musical learning has emerged as a possible intervention due to the similarities between musical learning–a non-verbal language–and verbal language itself. In particular, musical learning can assist in the processing of lexical skills [5] and in improving pitch discrimination abilities in both speech and reading among non-musician children [6]. Cross-sectional studies have shown that the detection of pitch patterns (global structure) is predictive of performance on measures of phonological skills and reading ability [7]. Meanwhile, the structural development of the auditory cortex is influenced by early musical experience [8]. Additionally, it has been pointed out that a link exists between musical abilities and phonological skills [9]; however, the bases of these links are not clear [10].

The explanation of the causal paths to reading development via musical training may be referred to as “transfer” [11], [12]. The connection between musical learning and improving reading skills would be a “far transfer” because musical learning is not directly related to reading. Musical training is based on teaching and constant practice of non-verbal structures such as classical sheet music, while reading is verbal. An example of a “near transfer” would be learning to play a musical instrument and consequently developing motor skills.

Neuroimaging studies have shown that some cognitive functions, such as the ability to organize isolated words into meaningful sentences and the ability to organize a variety of musical notes into a melody, may involve common neural pathways for both speech and music [13].

Music education classes involve different cognitive functions that require complex auditory pattern-processing mechanisms, attention, memory storage and retrieval, motor programming, and sensory–motor integration [14]. However, a recent systematic review of the effectiveness of music education used terms including “dyslexia” and “reading difficulties/disabilities” and returned 876 citations, from which no randomized clinical trials (RCT) were found. Therefore, despite the fact that musical learning is popular and considered to be a beneficial intervention, there is no evidence from randomized controlled trials that demonstrates the potential advantages of music education on reading skills and consequently on academic achievement [15].

This research used a pragmatic RCT to address the effectiveness of music education for improving reading skills and academic achievement in children with reading difficulties, aged eight to 10. The main idea behind this pragmatic RCT was to reflect the heterogeneity of children with reading difficulties in the general public education system, minimizing, as a consequence, exclusion criteria and providing a more realistic scenario due its good external validity (generalizability of the results) [16].

The study aimed to test the effectiveness of music education classes for improvement of academic achievement (based on Portuguese and math grades) and word-level reading skills among children with reading difficulties. This trial is registered at ClinicalTrial.gov under the number NCT01388881.

## Method

### Recruitment

#### School selection - inclusion criteria

Two Brazilian non-governmental organizations, or NGOs (specifically, Partnerships of Education and Rukha’s Institute) that worked in impoverished neighborhoods in Sao Paulo city (e.g., in slums) assisted in selecting 10 public schools on the outskirts of the city. These schools were chosen based on several logistical and social factors:

- At least, one room available for music lessons. This room would also be needed for the team of psychologists, audiologists, and ophthalmologists to evaluate the children during the screening process and outcome assessments;
- The schools lacked music lessons in the curriculum.

#### Children’s selection - inclusion criteria

Teachers from the second to the fourth grades of these schools were asked to complete the Scale of Assessment of Reading Competence by the Teacher (EACOL) which contains 27 dichotomous items with good divergent and concurrent validity, evaluates the loud (17 items) and silent reading abilities (10 items) of elementary school children [17]. EACOL has a range of 29 to −29 points, where values closer to 29 represent a good reader, and the following cut-off scores were used to separate students into three categories: the poor reader (<−14.5), not-so-good reader (from 14.5 to −14.5), and good reader (>14.5).

The following instructions were given: “…for the children in your class with a reading ability below the mean for the corresponding grade, please fill out the EACOL.” A total of 733 EACOLs from 48 teachers were returned, but only 617 were considered valid. EACOLs were omitted if items were filled out inadequately– for example, there were more than two missing items or sequential answers in a single category, or teachers answered “yes” to all 27 items or “no” to all 27 items. The 617 valid children formed what we labeled the Sao Paulo Screening Sample (SP-Screening). On the basis of the SP-screening, the psychologists ranked the children who were classified as poor readers or not-so-good readers in order to identify a minimum of 24 and a maximum of 27 children with reading difficulties to participate in the (RCT) from each school. Because the 10 schools differed in their numbers of enrolled children, four schools did not meet the minimum criteria. In the other six schools, where the numbers of eligible children exceeded 24, a total of 27 names were randomly selected via a lottery. We allocated a maximum number of students in order to prevent likely dropouts during the academic year or loss due to exclusion criterion, which is described below.

After identifying the eligible children via the EACOL, the research team contacted the parents via a letter that described the objectives of the trial. The letter explained the study' aims, procedures, measurements, avoiding technical scientific vocabulary; together with it, it was requested the parents’ written informed consent which was approved by Ethical Committee from Federal São Paulo University (CEP0433/10) for their children’s participation. The Ethical Committee from Federal Sao Paulo University approved this consent procedure. Only the children whose parents gave the written consent were included in the study. All written informed consents were stored in the department of Psychiatry at São Paulo Federal University. This study was approved by the Ethical Committee from Federal São Paulo University.

#### Children’s selection - exclusion criterion

- To avoid bias related to cognitive problems, the included children were tested for non-verbal intellectual ability using the Raven’s Coloured Progressive Matrices [18], and children with scores below the 25^th^ percentile were excluded;
- To avoid confounders due to contamination or overlap of interventions, parents were asked if their children already were receiving any regular hearing or speech therapy and/or music classes (such as private music classes, social projects involving musical learning, or other music school experiences). Children participating in such programs were excluded from the study.

### Sample Size

In total, 240 children were eligible for the study after being chosen by their teachers; selected by the psychologists as having the worst reading scores; and authorized by their parents to participate in the study. This value was based on the sample size calculation, with the following points taken into account:

- the cluster two-level structure (i.e., children who are nested in the schools);
- the necessary number of children in each of the 10 schools selected to achieve the minimum statistical power (1−β) of 0.75;
- two measures (pre- and post-test assessments of the primary outcome); and
- the following parameters: ρ (rho – expected intraclass correlation coefficient (ICC)) = 0.025; the expected moderate effect size (δ = 0.45); α = 0.05; and J (number of clusters) = 10.

The number of children per school was 24, with 240 children in the total sample. From these 240 children, three were excluded because their parents retracted consent after the full assessment of primary and secondary outcomes, and two changed schools before the full reading evaluation took place. The in-cluster structure is also in accordance with pragmatic design, reflecting the reality of the educational system. Ultimately, a sample of 235 children (girls = 38.3%) with an average age of 9.15 years (SD = .05) was obtained from the SP-screening. The description of the above cited process can be found in the flow chart diagram.

### Measures

#### Potential confounders

Before the assessment of the primary and secondary outcomes occurred, the following were assessed in order to avoid confounders:

- The visual acuity of the children (age-appropriate) under conditions of monocular viewing, conducted by an ophthalmology technician using Snellen’s chart. The children were classified as either having visual alterations or not. Also, auditory processing was evaluated via the Simplified Auditory Processing Test (SAPT) [19] by a hearing and speech-language pathologist. The following auditory abilities were tested: sound localization in five directions; verbal and non-verbal sequential memory; and the elicitation of the auropalpebral reflex through instrumental sounds. The children were classified as having or not having problems in central auditory processing.
- The intelligence quotient (IQ) was measured by a trained psychologist using the complete Wechsler Intelligence Scale for Children–Third Edition (WISC-III) [20], [21].
- School background variables were collected, including the number of classmates of each included child and the annual presence of children in official classes.

#### Primary and secondary outcome

To measure children’s’ ability to analyze metaphonological skills, the Test of Phonological Awareness [22] was utilised. It consists of 10 subtests, each one featuring four items used to verify synthesis, segmentation, manipulation, syllabic transposition, phonemic synthesis, rhyme, and alliteration. Therefore, the score range was from 0 to 40.

Phonological awareness strongly predicts reading skills [23] and is widely accepted to be an area of deficit among dyslexic children [24], [25]. Reading is a complex and multivariate process, and so we focused on variables related to lower-level cognitive skills (word-level reading) as our primary outcomes. The measured skills included the following:

- A word accuracy task (rate of correct real words read per minute),
- A non-word accuracy task (rate of correct non-words read per minute) and
- An in-text accuracy task (rate of correctly read words per minute in the text).

The lists were used for the first time in this trial and included 88 words and 88 non-words. The words varied in occurrence frequency (high- and low-frequency words), bi-directional regularity (regular and irregular words according to grapheme-phoneme/phoneme-grapheme correspondence); and length (short, medium, and long words, as measured by the number of letters). The non-words were built with the same orthographic Brazilian Portuguese structure, and the same length of stimuli was used in the list of words. Psychometrically, the word and non-words tasks showed excellent indices, presenting high correlations (r = 0.92, p<0.001). In addition, both were correlated positively and moderately with phonological awareness (r_ word accuracy_ = 0.40 and r_ non-word accuracy_ = 0.37). As expected, the general Intelligence Quotient (IQ) was related poorly to word accuracy (r = 0.168; p = 0.01) and not correlated with non-word accuracy (r = 0.01; p = 0.131).

Regarding the text-reading task, three different texts were selected for the three different age groups. The baseline in-text accuracy correlated highly with word accuracy (r = 0.916; p<0.001) and with non-word accuracy (r = 0.873; p<0.001).

In all of the above situations, the children’s reading was audio-recorded for accuracy analyses. The researchers had intended to blind the speech-language pathologists who collected the primary outcome data, but during the second evaluation, comments about the study allocation from teachers, directors and from the own children make the speech-language pathologists discover about the status of school as intervention or control.

The secondary outcome was academic achievement based on Portuguese and math grades. These were measured four times by the teachers during the school year, which begins in February and ends in November. The school directors were contacted at the end of school year to collect the Portuguese and math grades from the children in the trial. The grades were measured from 0 to 10, with 10 being the highest possible grade. None of the school directors or teachers were blinded to the randomization status of the school.

### The Randomization Procedure

In July 2011 (the middle of the school year in Brazil), the 10 directors of the 10 schools were invited to participate in a lottery. Two opaque boxes were used: The first contained balls containing ordinal numbers from one to 10. The numbers that the directors picked corresponded with the sequence of the subsequent lottery. The second box contained five balls printed with the word “intervention,” and five others were printed with “control.” In a sequence determined by the ball number picked in the first lottery, each director was called to pick one ball from the second box–either a “control” ball or an “intervention” ball. For example, the director who picked the ball with the number five in the first lottery was the fifth to pick either a control or intervention ball from the second box. Because we worked with a purposeful sampling of the schools, the randomization procedure was important for excluding bias related to school selection.

### Intervention

Music education (briefly defined here as a process of musical learning) was methodologically and educationally based on Brazil’s National Curriculum Parameters (NCP) [26]. This program focuses on a modern approach to music education in which the process of musical learning is not restricted to the domain of Western and classical sheet music reading or to a high aptitude for a particular musical instrument. Rather, the program focuses on musical improvisation, composition, and interpretation in accordance with the National Association for Music Education [27].

Children were encouraged to create their own music and to perceive and identify musical elements (rhythm, melody, harmony) during 50-minute activities that occurred three times per week for five months starting at the end of June 2010 and ending the last week of October 2010. Children were called to create and play music as well as to explore the sounds and history of non-traditional classical instruments made for avant-garde musical compositions and composers of the 20th century. Each school received soprano and contralto block flutes, keyboards, and two music teachers.

All music teachers followed the same syllabus and musical activities to avoid educational bias and to make the classes as similar as possible. The teachers were randomly allocated to the five intervention schools. Every two weeks during the intervention period, supervisions were arranged with the researchers, who systematically verified whether the music teachers were following the NCP’s assumptions and educational structure. Two teachers were provided per class to improve children’s level of attention and to guarantee that if any music teacher was absent, the other would follow the pedagogical plan. To provide a realistic and naturalistic scenario, the control schools were not encouraged to offer musical activities. This measure was in consonance with the logical perspective of pragmatic RCTs which may not employ placebos [16].

Music education is a complex intervention, mainly in an educational RCT context. For example, it is impossible to standardize a day-by-day routine, as each class has a different reality, and the music education might involve a huge spectrum of activities. These activities include singing, exploration of rhythm (via corporal movement or corporal percussion), and instrumental practice (which could be the highly technical learning of a specific musical instrument, or using the instrument in an informal manner) [15]. All of the procedures and activities described above are intended to: a) try to systematize the same intervention based on the NCP, or b) try to provide the same quality of intervention across various settings. Even with traditional educational methods such as Kodaly (Hungarian method) or Orff (German method), day-by-day programs are not established.

### Description of Blinding

This RCT is an open label because the children who were selected for the intervention knew that they were receiving music classes. At the same time, the selected intervention schools (and their scholar communities, i.e., teachers and directors who were responsible to collect the secondary outcomes) knew about the children who were allocated to receive intervention.

### Statistical Analysis

Two different types of analyses were used to evaluate the effectiveness of the music classes. The first (and standard) method was intention-to-treat (ITT), an approach that assumes that every child in the intervention schools actually received the music classes [28]. The other method, CACE estimation method took into account the compliance status (children’s adherence to the music classes) [29], [30]. The compliance status is defined here as at least a 1% presence in the music classes during the five months because with a presence of less than 1%, we are considering children who are never-takers. CACE estimation, therefore, provides a realistic effect. Due to institutional, organizational, and schedule differences (i.e., start and end of vacation period, holidays, children’s regular examination period), the five intervention schools had different gross numbers of musical classes (two schools had 57 musical classes, one 55, and another 50). Therefore, in order to take these differences into account in the CACE analysis, we considered the percentage as a reference, instead of the gross number, to calculate the compliance criteria.

Following the CACE estimation method, we have considered these assumptions: 1) the treatment assignment is random (as described above); 2) potential outcomes for each child is unrelated to the treatment status of other individuals; 3) for never-takers (children who do not receive the music classes even if they were assigned to this extra-curricular activity) and always-takers, the distributions of the outcomes are independent of the treatment assignment; 4) there are no defiers (children who do the opposite of what they are assigned to do); and 5) the average causal effect of the treatment assignment on the treatment received is not equal to zero [31].

Although there is a practical issue motivating the using of cluster structure, a statistical advantage exists in this design: It is very likely that individual interaction exists between children from the same school conditions, which leaves the treatment condition (control or intervention) less likely to be contaminated by other conditions. Therefore, comparison of different conditions will be more valid [32].

The type of baseline distributions for the primary and secondary outcome variables were considered (zero-inflated, normal, gamma). In addition, the standard errors were adjusted for the survey design (i.e., taking the clusters into account), thus generating robust standard errors (RSEs). Baseline significances tests comparing children from control and intervention schools on its outcomes (primary and secondary) and on potential confounders were conducted via t-Student or Mann-Whitney tests for continuous outcomes (depending on its variance homogeneity and normality distribution) and, for binary outcomes it was used the Chi-square.

Considering the CACE estimation method and ITT analysis, the primary outcome was controlled by the confounders (visual acuity and central processing assessment, IQ, and so on) along with age, gender, and baseline values from the same outcome (i.e., word accuracy was controlled by word accuracy at baseline); the only exception was adding the model involving phonological awareness as an outcome, as visual acuity was not included.

A linear growth model was built for the ITT analysis of Portuguese and math grades through the school year; for the CACE, linear growth mixture modeling was used, allowing the incorporation of latent groups (complier and non-complier). Mplus version 6.12 was used to build all regressions and general mixture models.

## Results

As suggested by CONSORT [28] the participants’ flow chart is described in Figure S1 and baseline measures comparing intervention and control schools with its respective significances tests are described in Table 1.

**Table 1:** Comparisons (absolute values or means with theirs Robust Standard Errors) between control and intervention and its respective significance test.

Variables at baseline	Intervention Schools	Control Schools	*p-value*
Number of schools	5	5	
Number the children	114	121	
Number of children (max) per school	27	27	
Number of children (min) per school	17	23	
Mean of Accuracy of word (RSE)	9.44(0.82)	11.22(3.60)	0.79
Mean of Accuracy of non-word (RSE)	5.90(1.10)	5.16(1.42)	0.43
Mean of Phonological Awareness (RSE)	25.78(0.70)	23.95(0.70)	<0.001
Mean of Portuguese (mean and RSE)	4.35(0.10)	5.33(0.23)	<0.001
Mean of Math (RSE)	4.49(0.19)	5.5(0.27)	<0.001
Mean of IQ (RSE)	91.30 (3.35)	93.43(2.38)	0.88
Children with problems in SPTA	58	31	<0.001
Children with Visual Acuity Problems	47	33	0.02
Mean of number the children per class (RSE)	30.63(1.72)	31.11(2.46)	0.89
Attendance through scholar year (RSE)	188.08(1.38)	188.25(1.77)	<0.001
Drop out in the follow up	7	6	

Abbreviations: max = maximum; min = minimum; RSE = robust standard error.

As suggested by Assmann [33], in Table 1, we report a table of baseline data with an overall description of the characteristics of the patients rather than using significance tests. Although differences across groups at baseline were found, some authors pointed out that the use of significance tests for detecting baseline differences is questionable [34] and others that it is inappropriate [35], [36]. Senn argued that “this practice is philosophically unsound, of no practical value and potentially misleading” [35].

Considering ITT, accuracy of word (β = 2.57, p = 0.047) has shown to be marginally significant. This means children in the intervention school correctly read 2.57 words per minute more than children in other schools do. Also in the ITT estimates, the slopes of Portuguese (β = 0.21, p = 0.01) and math achievement (β = 0.25, p<0.001) were statistically significant for the intervention schools. This it means that every two measured months, children from intervention schools increased 0.21 in Portuguese and 0.25 in math grades. There was no observed improvement in phonological awareness (p = 0.35) and in-text accuracy (p = 0.23); non-word accuracy was negative and nonsignificant (β = −1.512, p = 0.40) (Table 2).

**Table 2:** Effects of Music Education considering ITT and CACE.

		Intention-to-treat (intervention vs. control)		Complier-Average Causal Effect (complier vs. non-complier)	
	Outcomes	Estimates	RSE	Estimates	RSE
(Reading)	Accuracy of non-word	−1.39	1.67	−1.3	0.959
	Accuracy of word	2.57*	1.29	13.983***	0.853
	Accuracy of text	3.00	2.519	0.41***	2.412
	Phonological Awareness	0.88	0.94	19.719***	1.00
(Portuguese Achievement)	Slope†	0.21**	0.076	0.77**	0.27
	Intercep	−1.00***	0.311	−1.07***	0.31
(Math Achievement)	Slope†	0.246***	0.062	0.491**	0.174
	Intercept	−0.004***	0.344	−1.253***	0.349

*p*-values are expressed as following: < = 0.05(*),< = 0.01(**), < = 0.001(***).

† Slope on school status (i.e., on intervention schools, in case of ITT analysis) and slope on CACE parameter (complier children, in case of CACE estimation).

RSE = Robust Standard Error.

Regarding estimates for the complier group (using the CACE estimation method, where comparisons are made considering the complier versus non-complier groups and the effect of the control group is fixed at zero), estimates of word accuracy, in-text accuracy, and phonological awareness are statistically significant; this means that complier children read 13.98 more correct words per minute than children who are non-complier. Indeed, positive slopes of Portuguese and math achievement showed to be statistically significant.

Comparing the CACE and ITT, the CACE estimates were mostly higher than those obtained using the ITT analysis (except for in-text accuracy and intercepts of math and Portuguese). RSEs were lower in the CACE estimation method for the primary outcome.

The ICC–the degree of correlation that is realized among outcomes of participants in the same cluster–for each primary and secondary outcome, the pre- and post-test results, and all respective standard errors and confidence intervals is shown in Table 3. There was a considerable loss in statistical power for phonological awareness variables due to an unexpectedly high degree of ICC variation. When the sample size was estimated, low values were expected (approximately 0.025). The ICC confidence intervals ranged from 0 to 0.596; the largest variation was observed in phonological awareness (lower bound = 0 and upper bound = 0.596).

**Table 3:** Intraclass correlation coefficient for primary and secondary outcomes at baseline and last assessment.

	Outcomes	Pre-test				Post-test			
		ICC	SE	95% CI		ICC	SE	95%CI	
Primary Outcome	Accuracy of word	0.18	0.08	0.01	0.34	0.22	0.1	0.03	0,41
	Accuracy of non-word	0.11	0.06	0	0.23	0.24	0.1	0.04	0,43
	Accurracy of text	0.12	0.07	0	0.26	0.15	0.08	0	0,30
	Phonological awareness	0.14	0.07	0	0.29	0.36	0.12	0.12	0,60
Secondary Outcome	Portuguese (baseline and forth assessment)	0.06	0.05	0	0.16	0	0.02	0	0,04
	Math (Baseline and fourth assessment)	0.2	0.09	0.02	0.37	0.75	0.05	0	0,18

Abbreviation: CI = Confidence Interval; SE = Standard Error; ICC = Intraclass Coefficient Correlation.

A positive growing slope (β = 0.77, p = 0.005) in Portuguese means that, every two months, the grades in Portuguese increased 0.77 points for the complier group when compared with non-complier children. Considering math (β = 0.49, p<0.001), each two months, the grades for the complier group increased 0.49, when compared with non-complier group. The statistically significant and negative intercept indicates that the Portuguese intercept for the complier group at baseline is 1.07 points lower than the non-complier group; in math, the complier is 1.25 points lower.

## Discussion

The ITT analyses were rather unpromising: There was only one marginal significant effect for the primary outcomes (accuracy of word reading) (p = 0.047), probably because if there is a real effect of music education, it could be attenuated among the children who were allocated to be in the intervention and have not taken it (absence in the music classes, or presence of less than 1%) and the children who had attained the music classes assiduously. However, taking into account complier status via CACE estimation, it is possible to observe more promising effects in all primary outcomes, in case of accuracy of word reading, it becomes 6 times bigger (from 2.57 to 13.98).

The only negative exception was for non-word accuracy, which was not statistically significant by either the CACE or ITT estimation. This finding may have resulted from the baseline rate of non-words per minute, which was superior in the intervention group using the ITT method (Table 1). Although in-text accuracy with CACE was lower than with ITT, it showed statistical significance for the former but not the latter, corroborating to the idea that when we consider CACE estimation the effects of intervention become more apparent.

The negative estimations (CACE and ITT) for non-word accuracy are not explained by the baseline differences between intervention and control schools (significance test showed p-value = 0.43) and were not significant at 0.05 for both analyses (for ITT, p-value = 0.40, and for CACE, the p-value = 0.18). Indeed, a possible interpretation for the unsettling negative value might be related to the automatized process of word-level recognition, which was assimilated by children from intervention schools (i.e., children from intervention schools performed better in word-accuracy tasks). Maybe children were reading non-words as words (i.e., the more rapidly automatized and more correct children read words, less precise they read the non-words because children may read non-words as words). However, this hypothesis was not our focus, and it might only be assessed via the evaluation of non-word reading task errors’ typology.

The positive slopes of Portuguese and math grades indicate that, throughout the academic year, children from schools allocated to be in the intervention (general effect via ITT analysis) and the complier children (CACE estimation) have trajectories that are not flat, being music education effective for improvement of academic achievement.

For the secondary outcomes, there was a higher probability of estimating the effects when considering a power of 0.8 because more than two assessments of Portuguese and math grades were collected. This effect formed a third level in the hierarchical model (first level formed by the child, second represented by the school, and third by the four equi-distant measures in grades throughout the school year). The ICC also was lower (ρ = 0.06).

Table 3 described different magnitudes of ICCs, which may be interpreted as the Pearson correlation coefficient between any two responses in the same cluster, measuring the degree of similarity among responses within a cluster [37]. High (and non-predicable) ICC values were obtained and directly influence our results; as a consequence, our statistical power tends to be reduced in outcomes where ICCs were inflated (i.e., in a general view, we underestimated the *ρ* value in the sample calculation [*ρ* = 0.025]). Values presented in Table 3 are important to guide future RTC research involving scholar populations with measures related to learning and reading abilities. However, the underlying reasons for variation between cluster will differ from trial to trial, but two points in a cluster randomized study, particularly one involving education strategies, might be addressed, as stated by Donner & Klar [38]: 1) the effect of personal interaction among cluster members who received the same intervention; and 2) the influence of covariates at the cluster level, where all individuals in a cluster are affected in a similar manner as a result of sharing exposure to a common environment.

Some important limitations must be highlighted. First, due to issues which are inherent to pragmatic RCTs, when ITT is estimated, control schools may not have an active placebo (i.e., a “non-active” or placebo program was not introduced). Consequently, part of the improvement in reading skills and in Portuguese and math grades, in the ITT analysis, could result from the attention the music teachers paid to the students. Various developmental antecedents (social deprivation, socioeconomic status, family size, maternal reading, a stimulating home environment, maternal depression, and child negligence) are small but significantly related to reading achievement [38].

Because our children came from impoverished neighborhoods in Sao Paulo city, they may be influenced by these non-measured developmental antecedent factors, and, as a consequence, the musical activity may have functioned in two different ways: 1) as a psychological effect due to the “extra” attention from music teachers, and 2) as an environmental effect due to the provided stimulation itself (e.g., dance classes also would provide perceptions of rhythm). Therefore, to argue that the development of musical perception skills can account completely for the improvement in reading and academic achievement would be misleading in this experiment. Furthermore, because musical perception skills were not assessed throughout the full longitudinal study, we cannot presume that the more musical skills, the better the improvement of reading skills in our population will be. However, this pragmatic RCT did not aim to evaluate what in music classes would improve reading and academic achievement, but to pragmatically evaluate the effectiveness of music education as an intervention for reading difficulties.

Considering estimates of CACE, considerations about placebo are irrelevant because, as it was pointed out about the CACE assumptions, the effect of the control group is fixed at zero. The focus was exclusively on the complier and non-complier groups that were compared with one another.

Lately, the reading measures also were limited to the decoding process and methaphonological skills (word-level reading skills); therefore, we did not study reading skills beyond word-level decoding, such as comprehension.

### Conclusion

Based on the ICCs obtained in this study, future researchers should consider at least 24 schools (12 intervention schools and 12 control schools) with 24 participating children per school in order to reduce issues with power due to high variations in ICC, as was observed with the phonological awareness variable. Increasing the number of children per class does not significantly solve the power problem. At the same time, this increase would likely make it more difficult for the music teachers to properly conduct the musical activities. If some effect “exists,” the number of schools must be increased in order to increase the degree of power in future research for outcomes with high ICC variations. In future models and exploratory trials, placebo interventions (e.g., cooking classes) also should be implemented, while measures related to developmental antecedents should be evaluated and used as covariates.

Despite the noted limitations, this first RCT about music education is pragmatic and showed promising positive effects on reading skills and academic achievement considering CACE estimation, corroborating the theoretical rationale behind the music-based intervention, which admittedly is an unorthodox approach (for details see [39]). However, before recommending music classes as a public policy, more investigation and data about the effectiveness of music education and theoretical models explaining the impact of music abilities on reading skills are necessary, particularly in countries/scholar populations with low estimates of reading performance and academic achievement, as well as high levels of disparity between public and private schools.

## Supporting Information

Figure S1 **Flow diagram.**
(DOC)
