# Supplementary material for: Effectiveness of Music Education for the Improvement of Reading Skills and Academic Achievement in Young Poor Readers: A Pragmatic Cluster-Randomized, Controlled Clinical Trial
Source: PLoS One. 2013 Mar 27;8(3):e59984. doi: 10.1371/journal.pone.0059984 (PMC3609825; doi:10.1371/journal.pone.0059984)
Supplement: Figure S1 — Flow diagram. (DOC) [file pone.0059984.s001.doc]

**Flow Diagram**

**Allocation**

**Analysis**

**Follow-Up**

**Enrollment**

Assessed for eligibility (n=240)

Excluded (n=5)

- Parents retracted consent (n=2)
- Declined to participate (n=3)

Analysed (n=114) in both analysis (ITT and CACE)
♦ Excluded from analysis, because we did not have the end point evaluation from them. (n=7)

Lost to follow-up (children who changed school) (n=7)

Allocated to musical classes (n=114)

♦ Considering ITT, received allocated intervention (n=114)

♦ Considering CACE, received allocated intervention (n=91)

Lost to follow-up (children who changed school) (n=6)

Allocated to control (n=121)

♦ Considering ITT, received allocated control (n=121)

♦ Considering CACE, for each outcome a latent group (non-complier) is calculated, being the *n* non-constant in the control

Analysed (n=115) in both analysis (ITT and CACE
♦ Excluded from analysis, because we did not have the end point evaluations from them (n=6)

Randomized (n=235)
